# Projection of the Effects of the COVID-19 Pandemic on the Welfare of Remittance-Dependent Households in the Philippines

**DOI:** 10.1007/s41885-020-00078-9

**Published:** 2020-09-25

**Authors:** Enerelt Murakami, Satoshi Shimizutani, Eiji Yamada

**Affiliations:** JICA Ogata Sadako Research Institute for Peace and Development, 10-5 Ichigaya Honmuracho, Shinjuku-ku, Tokyo, 162-8433 Japan

**Keywords:** COVID-19, Remittance, Migration, Philippines, Household welfare, F22, F24, O12, O15

## Abstract

The Coronavirus Disease 2019 (COVID-19) is inevitably affecting remittance-dependent countries through economic downturns in the destination countries, and restrictions on travel and sending remittances to their home country. We explore the potential impacts of the COVID-19 pandemic on the welfare of remittance-dependent households using a dataset collected in the Philippines prior to the outbreak. First, we confirm that remittances are associated with welfare of households, particularly for those whose head is male or lower educated. Then, we use the revision of the 2020 GDP projections before and after the COVID-19 crisis to gauge potential impacts on households caused by the pandemic. We find that remittance inflow will decrease by 14–20% and household spending per capita will decline by 1–2% (food expenditure per capita by 2–3%) in one year as a result of the pandemic.

## Introduction

The Coronavirus disease 19 (COVID-19) is a devastating pandemic with global effects and is undoubtedly one of the largest macro-level shocks to the world economy, as evidenced by the already ominous economic indicators. While the adverse effects on the economy are revealing at the macro-level, the impact of the pandemic is likely to be heterogenous across countries and individuals. Moreover, the adverse effects may not be confined to the domestic markets but may be transmitted internationally, particularly in the case of developing countries.

This paper explores the potential impacts of the COVID-19 pandemic on the welfare of households in a remittance-dependent country, which is likely to be severely exposed to external shocks. The pandemic is expected to substantially reduce the amount of remittances that migrants from developing countries can send home. The World Bank estimates that global remittances will decline sharply by about 20% in 2020, the sharpest in recent history, and that remittances to low and middle-income countries are projected to fall by 19.7%.^1^ Many migrants may lose their jobs or be forced to accept lower wages due to lockdowns or oil price crashes in their destination countries (IOM, 2020); they may not be able to send remittances due to stringent movement restrictions and exclusion of money transfer service providers from the list of “essential services” (World Bank, 2020b). Furthermore, many intended migrants who had been preparing for their departure in the near future will be forced to change their livelihood plans for the coming years. In 2019, 80% of the world’s total remittances flowed to low-and-middle-income countries (World Bank, 2020c); therefore, the negative impacts of the COVID-19 outbreak may be more serious in developing countries whose citizens heavily depend on remittances from migrant family members.

The Philippines is a sensible case to study for several reasons. First, the country is one of the largest source countries for migrants in the world and is one of the most remittance-dependent, ranked fourth in terms of remittance inflow (Yang, 2011). The proportion of remittances relative to the country’s GDP was close to 10% (World Bank, 2020a, b, c and d). Moreover, some of the countries that host Filipino migrants are the most seriously affected by lockdowns and oil price crashes. The number of overseas Filipino workers was estimated at 2.2 million in 2016 with the top destinations being Saudi Arabia, the United Arab Emirates, Kuwait, Qatar, Hong Kong, and Singapore, which combined accounts for two-thirds of total destinations (Philippine Statistics Authority, 2017). The diversity of destinations implies that the impact of COVID-19 may be heterogenous even among Filipino migrants. The Philippine Government has reacted by providing cash relief to overseas migrant workers and their families who are suffering hardship.^2^

In this paper, we use a household-level dataset which was collected in in the Philippines before the COVID-19 outbreak. We first pin down the empirical relationship between remittance income and welfare of households by two-stage least squares (2SLS) instrumenting remittance income by a macroeconomic variable exogenous to households. We then project the potential impact of the COVID-19 shock in destination countries on the welfare of remittance-dependent households by utilizing the revision of the 2020 GDP forecasts by the International Monetary Fund (IMF) and the World Bank, which were made before and after the outbreak of the COVID-19 pandemic. Taking the difference between the predicted outcomes of with- and no-COVID projections provides us with the potential shocks on the remittances and other economic welfare outcomes of remittance-receiving households. Our projections show that remittance inflow will decrease by 14–20% and household spending per capita will decline by 1–2% in one year, as a result of the pandemic. Furthermore, the negative impact can substantially vary across different type of households.

This paper proceeds as follows: Section 2 describes the dataset used in this study. Section 3 examines the effect of macroeconomic shocks on household living standards prior to the COVID-19 outbreak. Section 4 performs several projections to gauge the impact of the pandemic on household welfare. Section 5 concludes.

## Data Description

**Fig. 1:**
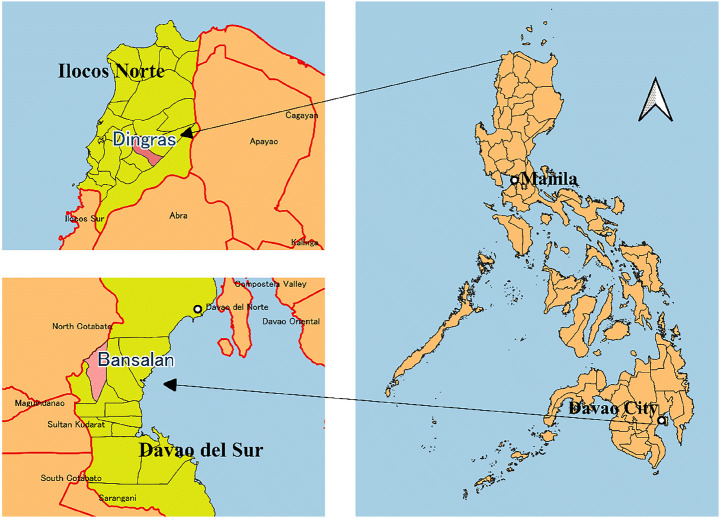
**Location maps of two municipalities.** Source: Generated by the authors based on GDAL’s administrative boundary shapefiles

This study utilizes the data from “Survey on Remittances and Household Finances in the Philippines,” conducted by the Japan International Cooperation Agency (JICA) in two rural municipalities in the country: Dingras, Ilocos Norte located in the Northern Luzon Island and Bansalan, Davao del Sur located in the southern island of Mindanao (Fig. 1).^3^ The survey is constituted of two rounds of data collection. The first-round survey was conducted between August and September 2016. The sample size was 834. The second-round survey was implemented between June and August 2017. The sample size was 668.^4^ The target sample size at the first-round was 200 overseas migrant households and 200 non-overseas migrant households in each municipality, which were randomly selected in each area. A migrant household is defined as a household which had at least one member who permanently resides or used to reside in this household but is now currently working or living overseas. Given that the stock of overseas Filipino was about ten million in 2013 (Commission on Filipinos Overseas, 2013), migrant households were oversampled. A total of 2429 overseas migrant households and 5172 non-overseas migrant households were listed in Dingras while a total of 563 overseas migrant households and 19,797 non-overseas migrant households were listed in Bansalan. Next, stratified random sampling was carried out for each municipality. The barangays within each municipality served as strata and the sample households were randomly selected within each barangay.^5^ The sample of 200 overseas migrant households was proportionately distributed among the barangays. Once the number of overseas migrant households was allocated among the barangays, an equal number of non-overseas migrant households was selected within each barangay.

Table 1 reports the summary statistics of the variables.^6^ In order to investigate the impact of the crisis on remittance-dependent households, we limit our sample to only those households that reported receiving remittances in at least one survey rounds. Columns (1)–(3) report the summary statistics for those remittance-receiving households and Columns (4)–(6) show those using all the households in our sample. Remittance-receiving households spend more both in terms of food and non-food items. They also make more savings and less loan payment on average. They earn less from agricultural and nonagricultural work and domestic sources. “*ECON*” is a weighted average of destination and home countries per capita GDP, and is explained in more detail in the next section. On average, heads of the households are 54 years old, and households are made up of 5 or more members, which includes overseas migrants. The education level of household heads is diverse. More than one-third completed only elementary school or have a high school degree; a quarter graduated college or higher education. The most common occupation among household heads is agriculture. Both the variables of education and those of occupation are binary (using non-educated or non-working heads of households as the reference).^7^

**Table 5:** **Comparison of percent distribution of the age and education attainments**

	(1)	(2)	(3)	(4)
**Characteristics**	**Males**		**Females**	
	Our Sample	2018 NMS	Our Sample	2018 NMS
**Age**				
0–14	27.4	31.4	23.0	30.4
15–30	25.4	27.8	24.7	26.9
30–45	20.5	19.9	20.7	19.9
45–60	15.7	13.6	16.6	13.8
60 or over	11.1	7.3	15.0	9.1
**Educational Attainments of all individuals above 5 years old**				
No education	5.9	4.0	2.7	3.8
Completed/attended elementary	29.5	35.2	30.9	30.9
Completed/attended high school	40.2	39.8	32.5	39.7
Completed post-secondary	0.0	2.6	0.0	2.4
Completed/attended college or higher	24.4	18.3	34.0	20.8

Note: Columns (1) and (3) show the distributions of individuals of the households in our survey sample. Columns (2) and (4) report the corresponding numbers obtained from 2018 NMS (National Migration Survey) of the Philippines (PSA and UPPI 2019)

## Empirical Analysis

We aim to measure the impact of the macroeconomic conditions in the destination countries on the outcomes relating to household living standards through remittances. There is a concern about the endogeneity issue since household welfare outcomes are likely to be affected by remittances and vice versa. It is well known that addressing endogeneity is one of the most crucial elements of estimation relating to remittances and the effects (McKenzie et al. (2010)). This is an important issue for our estimation by pooling observations rather than using panel fixed effects to remove latent characteristics of the sample households. In the context of the Philippines, remittances are often motivated to finance non-food consumption in the Philippines, which makes the OLS estimate on non-food consumption biased (less problematic for food consumption). This may be the case for flow of assets too. Moreover, remittances are substitute for domestic income but a third factor like endowment may make the estimate obscure since high endowment migrants holds higher ability to earn domestically.

Thus, we employ a two-stage least squares (2SLS) estimation using an index of the macroeconomic performance of the destination countries as an instrumental variable.^8^ We construct the “economic performance (*ECON*)” variable by taking the weighted average per capita GDP of the country of residence of each household member, including overseas migrants. More specifically, the “*ECON*” variable is constructed as:

$$ {ECON}_{it}=\ln \frac{\sum_{k\in \mathcal{K}(i)}{g}_{kt}\times {n}_{kit}}{\sum_{k\in \mathcal{K}(i)}{n}_{kit}} $$

Here, $\mathcal{K}(i)$ refers to the set of countries where the members of household *i* live, *g*_*kt*_ is the log GDP per capita in country *k* in *t* (2016 or 2017), and *n*_*kit*_ is the number of household *i*’s adult member who live in country *k*.^9^

We assume that GDP per capita is exogenous to the amount of remittances in each household. Our assumption means that *ECON* picks up supply-side shocks on migrants’ remittances, which reflects labor market conditions that they are exposed to in the destination countries. We acknowledge the possibility that our instrumental variable can also be correlated with demand-side shocks that would cause biases of the coefficients. Specifically, it might be the case that household’s latent characteristics and the choice of destination are closely associated; high endowment migrants are also likely to choose a high-income destination country, which could result in overestimation of the coefficient on the remittances. We also notice that it might be hard to establish exclusion restriction here since changes in economic performance outside the Philippines will have direct effect on household welfare in the country not through remittances but trade and financial channels affecting wage and employment prospects.

In the estimation, we use a level specification by pooling the observations at the first and second rounds, rather than a fixed effect model to remove unobserved heterogeneity. The main reason is to utilize a larger variation in the amount of remittances, the main variable, to obtain stable estimation results. Since the survey interval is short (less than one year), we see little change in the amount of remittances during the survey period. Instead of utilizing a variation between two periods in the same households, we pooled the data at both baseline and endline. The advantage is we can obtain a larger variation between households while the disadvantage is to not able to use a fixed effect model but the cost is abbreviated to some extent if we use a valid instrument.^10^

In the first stage, we regress the amount of remittances on the logarithm of the “*ECON*” variable and other covariates.

1 $$ {REMITTANCE}_{it}={\beta}_0+\beta \left({ECON}_{it}\right)+\boldsymbol{\gamma} {\mathbbm{X}}_{it}+ baranga{y}_i+{\lambda}_t+{\epsilon}_{it} $$

where *i* indexes households, and *t* refers to the survey round with 0 indicating 2016 and 1 indicating 2017. *REMITTANCE*_*it*_ is calculated as the monthly average either over the past 12 months for the first-round or for the period since the first-round visit in the case of the second round.^11^$\mathbbm{X}$ is a vector of household characteristics that were reported in Table 1. We also include barangay fixed effect (*barangay*_*i*_) and survey round fixed effect (*λ*_*t*_). Lastly, *ϵ*_*it*_ is a well-behaved error term. This specification exploits cross-country variations of GDP per capita to explain variations in the amount of remittance across households, rather than exploiting within-household variations of remittances between the two survey rounds.

Column (1) of Table 2 shows the results of the first stage regression. We performed a weak IV test and confirmed that F-test statistic for weak IV is 137.48 with *p* value of 0.00. The coefficient on “*ECON*” is positive and significant and indicates that a 1% increase in “*ECON*” leads to a 1.67% increase in income from remittances per capita; this implies that a significant economic recession in the destination countries will lead to a substantial drop in remittances.

**Table 1:** Summary statistics

	(1)	(2)	(3)	(4)	(5)	(6)
	Households Receiving Remittances			All households		
Variables	N	mean	s.d.	N	mean	s.d.
log per capita household expenditure	760	8.314	0.975	1296	8.123	0.968
log per capita food expenditure (monthly)	760	6.924	0.869	1296	6.806	0.844
log per capita non-food expenditure (monthly)	760	7.874	1.168	1296	7.649	1.173
log per capita new saving deposit	760	0.842	1.915	1296	0.809	1.856
log per capita loan repayment	760	1.227	2.656	1296	1.315	2.719
log agricultural income	760	3.624	3.532	1296	3.811	3.464
log non-agricultural income	760	4.046	3.666	1296	4.212	3.556
log per capita income from domestic sources	760	6.192	2.855	1296	6.463	2.451
log per capita remittance income	760	6.067	2.534	1296	3.558	3.563
Destination per capita GDP (*ECON*)*	760	9.036	0.753	1296	8.605	0.772
Head’s age	760	53.79	14.27	1296	51.73	13.85
Head’s sex (=0 if female, = 1 if male)	760	0.668	0.471	1296	0.731	0.444
HH size including overseas members	760	5.268	2.283	1296	4.945	2.143
Head’s educational attainment						
Elementary	760	0.368	0.483	1296	0.407	0.492
General High School	760	0.342	0.475	1296	0.349	0.477
Technical Vocational	760	0.0474	0.213	1296	0.0409	0.198
Post Secondary	760	0.0105	0.102	1296	0.00926	0.0958
College or more	760	0.226	0.419	1296	0.185	0.389
Head’s occupation						
Manager	760	0.0316	0.175	1296	0.0293	0.169
Professional	760	0.0263	0.160	1296	0.0262	0.160
Clerical	760	0.0158	0.125	1296	0.0147	0.120
Service	760	0.0816	0.274	1296	0.0903	0.287
Agriculture	760	0.262	0.440	1296	0.271	0.445
Production	760	0.0184	0.135	1296	0.0231	0.150
Municipality (=0 if Bansalan, = 1 if Dingras)	760	0.521	0.500	1296	0.486	0.500

Next, we use the estimated dependent variable of remittances at the second stage regression.

2 $$ {Y}_{it}={\beta}_0+\beta \left({\overline{REMITTANCE}}_{it}\right)+\boldsymbol{\gamma} {\mathbbm{X}}_{it}+ baranga{y}_i+{\lambda}_t+{\epsilon}_{it} $$

The dependent variables *Y*_*it*_ are a logarithm of (1) average monthly household expenditure per capita, (2) average monthly household food expenditure per capita, (3) average monthly household non-food expenditure, (4) average monthly new savings deposits per capita, (5) average monthly loan repayments per capita, (6) agricultural income, (7) non-agricultural income and (8) average monthly household incomes from domestic sources.^12^ The main explanatory variable ${\overline{REMITTANCE}}_{it}$ is the log average monthly overseas remittance income per capita, which is projected by the first stage estimates.

Columns (2)–(9) of Table 2 convey the second stage results. We will focus on the coefficient on the logarithm of remittance income per capita, the main explanatory variable. The coefficient on the remittance income is positive and significant for household spending per capita and the size is 0.084 (Column (2)), showing that a 1% increase in remittance income is associated with a 0.08% increase in per capita household spending. When we split household expenditure into food and non-food spending, the coefficient is significant and larger for the former (Columns (3) and (4)), showing that a 1% increase in remittance income is associated with a 0.14% increase in per capita food spending. The coefficient is positive for new savings and negative for loan repayments, but it is not significant (Columns (5) and (6)). While the coefficient on agricultural income is not significant, it is negative and significant for non-agricultural income (Columns (7) and (8)). Income from domestic sources is negatively and significantly associated with income from remittances (Column (9)). Both coefficients on non-agricultural income and domestic source income are minus 0.22 and 0.23, showing that one fifth of a change in remittance income is abbreviated by those income under the market situation in 2016 and 2017.

Table 3 reports the estimation result by splitting the sample by type of head of household. We run the regression by subgroups to address heterogenous effect of remittances on welfare of households by sex, age, and educational attainment of the head of household. First, we see that the coefficients on total, food and non-food spending are positive and significant for male headed households while the coefficient is positive and significant, and the size is larger on food expenditure for female headed households. A larger remittance income is negatively and significantly associated with non-agricultural income and domestic sourced income for male-headed households and with agricultural income for female-headed households. Second, if we divided the sample by whether the head of household’s age is greater than 52 years old, the median of the head’s age in our sample, the coefficient on spending is only significant for food expenditure by households whose head is older and remittance income is negatively associated with agricultural income and domestic income for those households. Third, when we divide the sample by the head of household’s educational attainment, the coefficients on household spending are positive and significant for households whose head completed less than secondary school.

**Table 2:** Estimation results

VARIABLES	(1)	(2)	(3)	(4)	(5)	(6)	(7)	(8)	(9)
	First stage	Second stage							
	log per capita remittance income	log HH expenditure per capita	log food expenditure per capita (monthly)	log non-food expenditure per capita (monthly)	log new saving deposit per capita	log loan repayments per capita	log agricultural income	log non-agricultural income	log Income from domestic sources
ECON (Instrumental Variable)	1.666***								
	(0.142)								
log remittance income per capita		0.0842**	0.137***	0.0642	0.112	−0.00301	−0.161	−0.232*	−0.224**
		(0.0365)	(0.0249)	(0.0437)	(0.0682)	(0.0656)	(0.114)	(0.130)	(0.0941)
Head’s age	−0.0184	−0.00374	−0.0288	−0.00327	0.0698	0.170***	0.107	0.0331	−0.0559
	(0.113)	(0.0221)	(0.0191)	(0.0248)	(0.0771)	(0.0612)	(0.0755)	(0.0891)	(0.0676)
Square of head’s age	0.000233	4.11e-05	0.000197	6.29e-05	−0.000643	−0.00138***	−0.000819	−0.000370	0.000510
	(0.000978)	(0.000195)	(0.000169)	(0.000222)	(0.000645)	(0.000525)	(0.000664)	(0.000793)	(0.000617)
Head is male	−0.441	0.189*	0.0244	0.265**	−0.241	0.249	0.280	0.141	0.212
	(0.394)	(0.101)	(0.0891)	(0.125)	(0.238)	(0.228)	(0.324)	(0.443)	(0.328)
HH size including overseas members	−0.00478	−0.101***	−0.0812***	−0.0988***	−0.0365	0.0133	−0.161**	0.292***	0.0230
	(0.0577)	(0.0239)	(0.0162)	(0.0293)	(0.0475)	(0.0490)	(0.0642)	(0.0670)	(0.0498)
Head’s educational attainment									
Elementary	1.398	0.526**	−0.176	0.914**	0.906	0.603	−0.117	2.175**	0.490
	(0.991)	(0.238)	(0.454)	(0.462)	(0.683)	(0.468)	(1.917)	(0.969)	(1.346)
General High School	1.087	0.557**	−0.229	0.995**	1.150	0.754	−0.805	2.377**	0.760
	(1.070)	(0.252)	(0.462)	(0.469)	(0.714)	(0.498)	(1.919)	(1.063)	(1.391)
Technical Vocational	1.262	0.949***	−0.169	1.402***	0.982	0.437	−2.029	3.848***	0.920
	(1.073)	(0.342)	(0.473)	(0.539)	(0.743)	(0.567)	(1.970)	(1.344)	(1.468)
Post Secondary	1.876	1.023*	0.128	1.430*	1.949*	−0.598	−1.453	1.903	−0.547
	(1.197)	(0.544)	(0.507)	(0.759)	(1.115)	(0.697)	(2.067)	(1.359)	(1.562)
College or more	1.728*	0.947***	−0.259	1.480***	1.405**	1.246**	−0.284	3.311***	1.298
	(0.962)	(0.251)	(0.459)	(0.475)	(0.716)	(0.493)	(1.921)	(1.053)	(1.379)
Head’s occupation									
Manager	1.301**	−0.00497	0.173	0.0500	0.111	1.037	0.358	3.325***	2.087***
	(0.598)	(0.194)	(0.175)	(0.234)	(0.946)	(0.880)	(1.098)	(0.630)	(0.577)
Professional	1.312*	0.616***	−0.191	0.705***	−0.220	1.265	0.207	1.935*	1.883***
	(0.794)	(0.216)	(0.465)	(0.271)	(0.458)	(0.974)	(1.069)	(1.146)	(0.448)
Clerical	0.319	0.720**	0.506	0.718*	−0.548	1.016	0.137	2.025*	0.902
	(0.916)	(0.294)	(0.316)	(0.387)	(1.084)	(1.091)	(1.496)	(1.218)	(1.104)
Service	0.451	0.518***	0.378**	0.556***	−1.710*	0.805*	−0.580	2.131***	1.629***
	(0.498)	(0.163)	(0.186)	(0.192)	(0.905)	(0.478)	(0.575)	(0.677)	(0.345)
Agriculture	−0.148	0.182	0.383***	0.120	−0.264	−0.239	2.437***	0.292	1.649***
	(0.356)	(0.129)	(0.103)	(0.150)	(0.348)	(0.272)	(0.376)	(0.454)	(0.292)
Production	3.087**	−0.269	−0.328	−0.126	−1.116*	0.606	0.251	3.150***	2.598***
	(1.379)	(0.288)	(0.381)	(0.279)	(0.660)	(0.676)	(0.727)	(0.862)	(0.979)
Constant	−8.792***	8.454***	7.863***	7.784***	−2.756	−3.281**	1.188	2.208	9.306***
	(3.081)	(0.805)	(0.767)	(0.945)	(2.054)	(1.653)	(2.651)	(2.610)	(2.229)
Observations	760	760	760	760	760	760	760	760	760
R-squared	0.476	0.355	0.291	0.319	0.193	0.444	0.487	0.409	0.319

*** *p* < 0.01, ** *p* < 0.05, * *p* < 0.1

In summary, the estimation results confirm that a decline in remittances discourages household spending per capita and is partly abbreviated by non-agricultural income and domestic income.

## Projections

To quantify the scale of the economic shocks caused by the COVID-19 pandemic on the relevant countries, we use the per capita GDP predictions available for each country in 2020 from growth forecasts by the International Monetary Fund (IMF) ‘s “World Economic Outlook” published in October 2019 (IMF 2019) and June 2020 (IMF 2020), and the World Bank (WB)‘s “Global Economic Prospects” published in January (World Bank 2020a) and June 2020 (World Bank 2020d).^13^ The IMF’s outlook from October 2019 and the WB’s outlook from January 2020 can be seen as a “no-COVID” forecast, which helps us to construct the hypothetical “*ECON*” variable, where a COVID-19 pandemic had not taken place. Conversely, the revised IMF’s outlook from June 2020 and the WB’s outlook from June 2020 can be used to construct the “with-COVID” economic scenarios that will affect remittances from migrant workers. The “with-COVID” projections contain two cases in the “World Economic Outlook” and three cases in the “Global Economic Prospects”. Details of the scenarios are given in Table 4. We implicitly assume that the change in the prediction of GDP for 2020 in the two different timings is entirely attributed to the pandemic.

**Table 3:** Subsample estimation results by type of head of household

	(1)	(2)	(3)	(4)	(5)	(6)	(7)	(8)
VARIABLES	log per capita expenditure	log per capita food expenditure	log per capita non-food expenditure	log per capita new saving deposit	log per capita loan repayments	log agricultural income	log non-agricultural income	log Income from domestic sources
All (Table 2)	0.0842**	0.137***	0.0642	0.112	−0.00301	−0.161	−0.232*	−0.224**
	(0.0365)	(0.0249)	(0.0437)	(0.0682)	(0.0656)	(0.114)	(0.130)	(0.0941)
Male head	0.0885**	0.123***	0.0708*	0.0802	0.0674	−0.0722	−0.357***	−0.219**
	(0.0358)	(0.0253)	(0.0429)	(0.0695)	(0.0726)	(0.121)	(0.127)	(0.0891)
Female head	0.139	0.229**	0.129	−0.157	−0.360	−0.926**	0.518	−0.0375
	(0.0934)	(0.0900)	(0.114)	(0.255)	(0.222)	(0.361)	(0.478)	(0.370)
Head’s age > 52	0.0784	0.169***	0.0490	0.148	−0.0372	−0.618***	−0.319	−0.506***
	(0.0526)	(0.0448)	(0.0637)	(0.121)	(0.127)	(0.185)	(0.224)	(0.194)
Head’s age < = 52	0.0683	0.0792	0.0612	0.0699	0.000399	0.334*	−0.203	0.185
	(0.0690)	(0.0638)	(0.0778)	(0.154)	(0.142)	(0.193)	(0.233)	(0.163)
High educated head	0.0710	0.147***	0.0546	0.118	−0.0392	−0.0893	−0.265	−0.228*
	(0.0554)	(0.0411)	(0.0638)	(0.103)	(0.0982)	(0.171)	(0.195)	(0.138)
Low educated head	0.150***	0.141***	0.148***	0.00480	0.0198	−0.331**	−0.0816	−0.151
	(0.0416)	(0.0345)	(0.0526)	(0.0782)	(0.104)	(0.134)	(0.171)	(0.138)

(Note) The coefficients on log remittance income per capita in each specification are reported

We compute the predicted values by plugging the hypothetical *ECON* variables, constructed using each of the GDP per capita forecasts for remittance-receiving households, into our 2SLS estimates that shows statistical significance in Table 2. We then compare the mean predicted values of with-COVID scenarios with that of no-COVID scenario in each growth outlook for the various outcome variables in each projection scenario. We do not consider the compensating effect of domestic income on the decline of remittances because the Philippine economy is also seriously affected by the pandemic.

Table 4 shows the potential impacts of the COVID-19 as percentage changes in the predicted remittances, expenditure and income under the with-COVID scenarios against the no-COVID scenario as per each growth outlook. The negative impact of the pandemic on remittances is serious, with a decline of as high as 14–20%, which is comparable with the World Bank’s forecast for decline in remittances in the East Asia and Pacific region in 2020. Moreover, our estimate is close the recently published ADB projection (Kikkawa et al. 2020) showing that the remittance to the Philippines will decline by 20.2%. The adverse effects are more pronounced under the “with-COVID scenario two” by the World Bank, while “with-COVID scenario one” by the IMF and “with-COVID scenario one” by the World Bank are closer in magnitude. The household spending per capita would decline by 1–2% in each scenario. Of the total spending, food expenditure has the highest drop by 2–3%. Thus, our predictions show that remittance inflow will decrease by 14–20% and household spending per capita will decline by 1–2% (food spending by 2–3%) in the space of one year during the COVID-19 pandemic. Reminding our subsample analysis in the previous section, households with male or low educated head will further decrease per capital expenditure while female headed households will see more substantial drop in food consumption due to the decline in remittances income.

Those projections must be understood in conjunction with several reservations. First, we use household data from heavily remittance-dependent regions that do not necessarily conform to the average in the Philippines. Second, our projection captures a short-run (during the year 2020) effect of the pandemic on household welfare but the negative impact would be more serious over a longer term. Third, we summarized all aspects of the virus outbreak into a change in per capita GDP. We may need to take a more nuanced approach using data on international restrictions on travels and remittance transactions. Fourth, we boldly sum up complex processes within a serial decision-making process carried out by households in relation to migration and remittances into the “amount of remittance”. Disentangling the effect of the pandemic over the migration process is an important agenda for future research. Fifth and lastly, we implicitly assume that an increase in remittances will have the same magnitude on household-level outcomes as will decreases in remittances associated with the pandemic. The symmetry assumption that the sensitivity of household-level outcomes remains the same during the pandemic should be examined by the actual post-pandemic data.

## Conclusion

Using a household-level dataset in heavy migrant-dependent regions before the outbreak in the Philippines and the 2020 GDP projections made by the IMF and the WB, we evaluated the potential impact of the COVID-19 pandemic. Our projection shows that remittance inflow will decrease by 14–20% and household spending per capita will decline by 1–2% (food expenditure per capita by 2–3%) in one year as a result of the pandemic.

The pandemic is still ongoing. Future research should use the actual data in migrant-sending countries after the COVID-19 outbreak to quantify the adverse effects on household living standards. While it is not easy to conduct a survey during the pandemic, this line of research will be very informative for future policy responses.

## Appendix

**Table 4:** Potential impacts on household welfare

	Percent changes, IMF		Percent changes, World Bank		
	With-COVID 1	With-COVID 2	With-COVID 1	With-COVID 2	With-COVID 3
Remittance receipt	−15.63	−14.64	−15.86	−19.91	−14.11
Total expenditure	−1.41	−1.31	−1.46	−1.86	−1.29
Food expenditure	−2.31	−2.14	−2.38	−3.03	−2.11
Nonfood expenditure	−1.08	−1.00	−1.11	−1.42	−0.98
New savings	−1.84	−1.71	−1.89	−2.43	−1.67
Loan repayment	0.05	0.05	0.05	0.07	0.05

IMF projections: Scenario “no-COVID”: the projection of GDP in 2020 as of October 2019

Scenario “With-COVID 1”: the updated baseline growth projections as of June 2020, assuming a slower recovery after the second half of 2020. Global growth declines by 4.9%

Scenario “With-COVID 2”: the updated alternative growth projections as of June 2020, assuming that the pandemic recovery is faster than the baseline projections of June 2020. Global growth declines by 4.4%

WB projections: Scenario “no-COVID”: the projection of GDP in 2020 as of January 2020

Scenario “With-COVID 1”: the baseline scenario in the WB’s June 2020 growth forecasts, assuming that the lockdown lasts until the end of the second quarter of 2020. The global output declines by 5.2%

Scenario “With-COVID 2”: the downside scenario, assuming that the lockdown lasts until the end of third quarter of 2020. The world GDP declines by 8%

Scenario “With-COVID 3”: the upside scenario, assuming prompt recovery after the second quarter of 2020. The world GDP declines by 4%

## Footnotes

1 https://www.worldbank.org/en/news/press-release/2020/04/22/world-bank-predicts-sharpest-decline-of-remittances-in-recent-history. The decline is projected to be 13% in the East Asia and Pacific region.

2 https://www.owwa.gov.ph/index.php/news/regional/85-1-600-active-owwa-members-in-davao-del-sur-receive-cash-relief-assistance-from-owwa-xi.

3 These municipalities were selected in order to oversample households with overseas migrants and provided the necessary collaboration with us to implement the survey and information for listing.

4 While we chose only two municipalities in the survey due to resource limitation, our study area covers regions with different characteristics in terms of dependency on migration and remittances. 32.3% of the households in Dingras has at least one migrant while only 2.8% in Bansalan (10.6% of the total samples of two municipalities). According to 2018 National Migration Survey (PSA and UPPI 2019), 8.9% of the households in the Ilocos Region (where Dingras belongs) have at least one OFW (Overseas Filipino Workers) in the past 12 months and 5.7% in the Davao region where Bansalan is located (6.4% nationwide). Although our sampling design does not generate nationally representative dataset, respondents in our sample are comparable to the 2018 National Migration Survey (NMS) of the Philippines (PSA and UPPI 2019). We compare the distributions of age and educational attainments between two surveys, and find that our sample individuals are slightly older and proportion of college attendees or graduates is higher. The detail is reported in Appendix Table 1.

5 The barangay is the smallest political unit and a subdivision of a city or municipality.

6 Per capita expenditure is systematically larger and the ages of the heads of household is higher for the attrition households at the first-round.

7 While the job of a seamen makes up a large part of the migrant job market, our sample does not contain many of these migrants.

8 In order to gauge the direction of the bias stemming from OLS estimates, we checked to compare the regression outcomes with non-instrumented as well as instrumented remittance values. When we compare those results, we see that the coefficient loses significance in nonfood consumption and the coefficient gains absolute size and significance in non-agricultural and total income from domestic sources. The OLS results are available upon request.

9 We share the spirit with Ratha and Shaw (2007) that used weighted value of destination GDP in cross-country estimating remittances inflow.

10 In addition, we use the sub-sample of households with migrants only. We believe that the most fundamental selection-bias in the decision of whether or not to migrate is well-addressed by this sub-sample strategy.

11 The qualitative results are not changed if the average over the past 12 months is used for the second round.

12 The denominator of all “per capita” variables from the household survey is the number of household members excluding migrating members.

13 The initial projection by the IMF after the pandemic was released in April 2020 and updated in June 2020.
